# Repostioning of Telemedicine in Cardiovascular World Post-COVID-19 Pandemic

**DOI:** 10.3389/fcvm.2022.910802

**Published:** 2022-05-30

**Authors:** Kamal Sharma, Zeel Patel, Smeet Patel, Kalpen Patel, Shweta Dabhi, Jinish Doshi, MohmadSabir Amdani, Darshini Shah, Dhyanee Patel, Ashwati Konat

**Affiliations:** ^1^SAL Hospital, Ahmedabad, India; ^2^Amdavad Municipal Corporation (AMC) Medical Education Trust Medical College, Ahmedabad, India; ^3^Smt. Nathiba Hargovandas Lakhmichand (N.H.L.) Municipal Medical College, Ahmedabad, India; ^4^Pandit Deendayal Upadhyay (P.D.U) Medical College, Rajkot, India; ^5^Gujarat Cancer Society (GCS) Medical College, Ahmedabad, India; ^6^All India Institute of Medical Sciences Jodhpur, Jodhpur, India; ^7^Department of Zoology, Biomedical Technology and Human Genetics, Gujarat University, Ahmedabad, India

**Keywords:** telemedicine, cardiovascular disease, COVID-19, advantages, challenges

## Abstract

**Background:**

During the COVID-19 pandemic, telemedicine is a quickest expanding service solution to provide improved access to sophisticated healthcare that is efficient, cost-effective, and time-consuming.

**Methods:**

This analysis is evaluated on the basis of several studies that look at the history, benefits, various techniques, challenges, uses, and impact of telemedicine in the treatment of heart failure and cardiac rehabilitation as during COVID-19 outbreak.

**Results:**

Patients avoided or refused medical treatment during COVID-19 pandemic despite the risk of illness and the threat of infections spreading. Telemedicine has become a non-traditional form of care delivery due to better access and high-end technologies such as virtual consultations, face-to-face video, smartphone visits, two-way text communication, distant patient history, and distal characteristic assessment. Remote monitoring can help manage cardiovascular disease risk factors and increase patient participation in blood pressure, heart failure data, and workout or other activity progress.

**Conclusion:**

Based on the findings of past studies, we can infer that telemedicine is still an emerging subject in the treatment and management of cardiovascular disease. Telemedicine and similar technologies will also revolutionize healthcare services by expanding their reach and providing a big pool of database for better research and analysis.

## Introduction

Telemedicine is the evaluation and clinical care over long distances leveraging telecommunications technology, allowing low-income populations to access high-quality healthcare (1). Information and Communication Technology’s (ICT) have the potential to resolve some of the obstacles that eventually created countries have in delivering appropriate, cost-effective, and enhanced health care services. Telemedicine is a type of information and communication technology (ICT) that is used to overcome geographical obstacles and improve access to health-care services. This is especially beneficial for developing nations’ rural and underdeveloped communities, which have historically restricted access to health care (2).

“The shipment of quality healthcare, where proximity is a crucial factor, by all healthcare providers employing information and communication technologies for the interaction of valid information for diagnosis, treatment, and preventive care and injuries, research and evaluation, and professional development of healthcare professionals, all in the preferences of advancing the health of individuals and communities,” according to the World Health Organization (WHO) (3). Telemedicine dates from the mid- to late-nineteenth century, with one of the first documented occurrences occurring in the early twentieth century, when electrocardiograph findings were sent *via* telephone wires (2).

High rates of cardiovascular disease (CVD) have been linked to difficulties in diagnosing and referring patients, particularly in rural areas and developing countries like India (4–6). According to various studies and statistics, the burden of CVDs is fast increasing, which usually reflects of coronary heart disease tripling in the last thirty years. In India, cardiovascular disease appears to have overtaken cancer as the major cause of mortality. According to a survey conducted in 45 localities in 2004, cardiovascular disease (CVD) was responsible for 32% of all deaths. Infectious diseases cost the lives of 13% of the population (7).

With 32 million diabetics, India will already be regarded as the world’s diabetes hotspot. By 2025, this number is expected to climb to 69.8 million. Hypertensive persons are anticipated to increase from 118 million by 2020 to 214 million by 2025. A huge disparity in health-care distribution is exacerbating the problem. Despite the reality that more than 75% of Indians live in rural areas, more than 75% of Indian healthcare professionals work in urban settings (8).

There is an expanding market for telemedicine linked facilities around the country as the ICT platform improves. Specialists may now acquire clinical data of patients along with imaging information including such X-rays, echocardiography, as well as other photographs from many modalities thanks to the growing usage of desktops, smartphones, and tablet devices to access the internet (9).

Since the 1980s, telecardiology has been used to consult with cardiologists while also transmitting electrocardiograms (ECGs). The need for tele-echocardiography is being driven by the continued and significant growth of echocardiography as the primary cardiac imaging modality (40%), which includes the usage of portable ultrasound scanners. High-resolution echocardiographic images can now be displayed on tablet computer screens to provide diagnosis and treatment guidance, thanks to the diagnostic quality of these devices’ screens. The purpose of this research is to look into the history, benefits, issues, applications, and influence of cardiac rehabilitation as during COVID-19 pandemic.

## Advantages

Telemedicine was established with the primary objectives of enhancing chronic disease care, particularly in crises, in remote locations or in regions wherein access to health care services is limited. The employment of increasingly new digital technological tactics has spurred all use of telemedicine as during multiple phases of disease far more consistently, just like during the SARS outbreak in 2003 as, later, MERSCoV in 2013, and currently during the outbreak of COVID-19. This revolutionary innovation patient care system includes a number of conveniences for both the patient and the medical professional, in addition to the traditional mode of health care delivery.

## Efficiency in Terms of Cost

Brouwers et al. (10) observed that a Tele-rehab III clinical trial, which included the ongoing analysis of data from the SmartCare-CAD research, showed similar cardiac healthcare cost benefits over the intervention period but no differences in Quality adjusted Life Years (QALYs) between groups. When Cardiac telerehabilitation (CTR) was used in conjunction to center-based Cardiac Rehabilitation in the Tele-rehab III study, QALYs enhanced and health-care costs decreased well over course of 2 years [a cost savings of €878 ($1003) for the intervention group].

The economic benefits were attributed to a difference in cardiovascular hospital readmission (32 with in experimental group vs. 60 in the control group), which could have influenced participants’ Quality Of Life. This discrepancy in rehospitalizations could be related to Tele-rehab III participants having a larger residual cardiovascular risk; as a result, participants in the intervention group may very well have benefited more from a longer CR program. Extended CR interactions may be useful and cost effectiveness in higher-risk people, according to the outcomes of the Tele-rehab III clinical study in high-risk individuals, it is both useful and cost-effective (10).

Wang et al. (11) revealed that neoplasms (19.4%), traumas (13.9%), and cardiovascular disorders (10.3%) were the 3 most prevalent diagnoses in a telemedicine deployment in China’s Western province. 4,772 patients (39.8%) had their diagnoses changed as a result of teleconsultations, while 3,707 patients (77.7%) had respective evaluations severely amended. Moreover, 6,591 (55.0%) of individuals had their therapy modified, with 3,677 (55.8%) of those modifications unrelated to changes in diagnosis. The telemedicine network saved $2,364,525 (if patients traveled to the hub) or $3,759,014 (if patients did not extend to the hub) (if patients did not visit the hub, and specialists visited the spoke hospitals).

When surveying the entire community, including both use amongst, total medical costs reduced by roughly 15% between 2019 and 2020, according to Weiner et al. (12).

## Time Saving

Telemedicine has now become a form of “advance triage,” or triaging patients prior they enter an emergency room, during the COVID-19 outbreak. Despite the fact that it is self-quarantined, simple telemedicine, also known as on-desired telemedicine, seems to have become a handy way for individuals to be evaluated. This type of triage keeps patients and healthcare personnel safe while obtaining patient-centered therapy (13).

When compared to the conventional method, the novel telemedicine service cut expenditures and transit time by 56 and 94%, respectively, according to a Bangladeshi study. According to the research, the percent of participants were delighted with the newly formed telemedicine service (14). Table 1 shows the comparison of different telemedicine cost-effectiveness studies from Jiang et al. (21).

**TABLE 1:** Comparison of different telemedicine cost effectiveness studies from Jiang et al. (21).

Technologies or devices for digital health intervention delivery	Country	Targeted disease	Time horizon	Intervention vs. comparator	Incremental cost-effectiveness ratio
Burn et al. (22) (Short message service)	Australia	Coronary heart disease	Lifetime	TEXT ME^a^ program vs. UC^b^	TEXT ME program dominated UC
Grustam et al. (23) (Telephone support)	United Kingdom	HF^c^	0 years	TM^d^ vs. UC	€12,479/QALY^e^
Grustam et al. (23) (Telephone support)	United Kingdom	HF	0 years	NTS^f^ vs. UC	€8795/QALY
Grustam et al. (23) (Telephone support)	United Kingdom	HF	0 years	NTS vs. UC	NTS dominated TM
Martín et al. (24) (Mobile apps)	Spain	HF	Not declared	CardioManager vs. UC	€9.303/QALY
Thokala et al. (25) (Telemonitoring)	United Kingdom	HF	30 years	STS HM^g^ vs. UC	UC dominated STS HM
Thokala et al. (25) (Telemonitoring)	United Kingdom	HF	30 years	TM vs. UC	£11,873/QALY
Thokala et al. (25) (Telemonitoring)	United Kingdom	HF	30 years	Structured telephone support with a human to human contact vs. TM	£228,035/QALY
Cowie et al. (26) (Telemonitoring)	United Kingdom	HF	10 years	CardioMEMS Vs. UC	£19,274/QALY
Sandhu et al. (27) (Telemonitoring)	United States	HF	Lifetime	CardioMEMS vs. UC	US $71,462/QALY
Schmier et al. (28) (Telemonitoring)	United States	HF	5 years	CardioMEMS vs. UC	US $44,832/QALY
Martinson et al. (29) (Telemonitoring)	United States	HF	5 years	CardioMEMS vs. UC	US $12,262/QALY
Healy et al. (30) (Wearable medical device)	United States	SCA^h^	5 years	WCD^i^ vs. discharge home	US $26,436/QALY
Healy et al. (30) (Wearable medical device)	United States	SCA^h^	5 years	WCD vs. SNF^j^	WCD dominated SNF
Healy et al. (30) (Wearable medical device)	United States	SCAh	5 years	WCD vs. in-hospital stay	WCD dominated in-hospital stay

*^a^TEXT ME, Tobacco, Exercise, and Diet Messages; ^b^UC, usual care; ^c^HF, heart failure; ^d^TM, telemonitoring; ^e^QALY, quality-adjusted life year; ^f^NTS, nurse telephone support; ^g^STS HM, structured telephone support with a human-to-machine interface; ^h^SCA, sudden cardiac arrest; ^i^WCD, wearable cardioverter-defibrillator; ^j^SNF, skilled nursing facility.*

## Challenges of Telemedicine

The global outbreak of COVID-19 has resulted in significant morbidity and mortality. According to Chinese epidemiological statistics, people with concomitant cardiovascular illness are more likely to have substantial acute respiratory syndrome coronavirus 2 (SARS-CoV-2) infection with potentially fatal results (15).

Concerns regarding access have stifled telehealth’s enormous promise for a long time. Patients who’ve been elderly, live in distant places without dependable connectivity, or have a poor level of education are more likely to be denied telehealth services. One such factor appears to be age. Patients beyond the age of 65 battled with telemedicine technology, resulting in fewer video visits (16).

Low financing is wreaking havoc on all developing-country programs, including those that aren’t directly related to health care. Upkeep costs for keeping the system operating after preliminary tests and enthusiasm are substantially higher in poor countries when compared to comparable prices in developed countries. Most rural locations have inadequate fundamental ICT infrastructure, making any form of link with remote hubs and facilities impossible. One of the most obvious causes of telemedicine system crash is a scarcity of ICT infrastructure.

Telemedicine services, according to regulatory agencies, must be adequately controlled both within and across countries. Even the best telemedicine system may result in a less effective system if connectivity to the remote medical center(s) is poor. A restricted system with a good link, on the other hand, can produce high-quality results. The other potential challenges are as mentioned below:

• Methodology of payment
• Confidentiality and security of patient’s data
• Access discrepancies
• Depersonalization
• Appropriate utilization and adoption of digital world

## How to Overcome the Challenges of Telemedicine

The ability to use the appropriate technology is an important aspect of the telehealth adoption process. To bring telehealth services online, facilities will need PCs, tablets, smartphones, and other technology. The advantages of employing current digital technology, such as speedier communication, automatic backups, and the capacity to communicate with patients on the road, should be widely understood by healthcare practitioners and personnel.

Digital Literacy is very important for the success of telemedicine. Patients and physicians should feel at ease with the technology; but, old age patients, may have difficulty using it. Providers can discuss this technology with their patients to determine whether they will use a telehealth program, which includes sending and receiving personal health information, responding to SMS, and setting up automatic reminders.

## Telemedicine Success During COVID-19 Pandemic

Throughout COVID-19, the primary goal of telemonitoring is to provide patients with a “health management strategy” that defines a personalized goal for each patient and changes attempts to keep the measured indicators as close to ideal as possible. Because of its novelty and wide range of potential applications, a clear demarcation of circumstances for how to employ telemedicine in short bursts has also proven difficult (17).

Telemedicine could be used to deal with patients who are isolated at home or at a hospital. In this case, telemedicine provides sufficient protection for both professionals and caregivers by limiting physical exposure to infectious patients to extremely non-deferrable crises (18).

Cardiovascular disorders, in particular, demand constant monitoring, putting both sufferers, and practitioners at risk of infection (19). With rapid developments in e-health technologies witnessed during epidemics and pandemics, remote monitoring has spread transcend emergency scenarios in this setting (20). The SARS-CoV-2 coronavirus pandemic has aided in the management of a variety of chronic disorders in this area.

Top five reasons to seriously consider TELEMEDICINE:

• Better accessibility
• Efficient care
• Millennial demand fulfillment
• Decreased absenteeism
• Improved patient satisfaction

Benefits for hospitals and healthcare professionals:

• Extend the reach and provide more healthcare services to people and connecting to underserved populations with boosting patient connections.
• Make better use of clinicians’ outpatient slots.
• Monitor and manage protracted patient care with ease.
• Assist doctors in addressing more patients.
• Reduce patient access and manpower constraints challenges
• Preventing the delay in the diagnosis of certain fatal diseases
• Prevent the spread of airborne illnesses (for example, COVID-19 disease)—easily maintain social distancing practice.
• Expanding the pool of possible patients, resulting in additional revenue streams to clinicians and hospitals.
• Providing more preventive care.
• Providing more affordable health care services.
• Appointment no-shows are becoming less common.
• The gratification of providing superior patient care.
• Relationships with patients have been strengthened

Benefits for patients:

• Not having to travel a considerable distance for appointments.
• Not having to pay as much, especially when compared to emergency department or usual walk-in outpatient services.
• Not having to worry about a lack of local specialists for unusual disorders.
• No need to wait for assistance from a qualified healthcare professional.
• Specialized therapy for unique medical disorders is also easily accessible.
• Bedridden individuals can receive treatment swiftly and conveniently from the comfort of their own home.

## Conclusion

According to the current research, the use of telemedicine has increased significantly during pandemic. As a result of recent research and technical breakthroughs, telemedicine seems to have become a critical asset in the treatment of chronic illness’, including both healthcare personnel and patients. Enhanced patient education, device accessibility, and improved connection are all key components in introducing telemedicine in impoverished nations.

## Author Contributions

All authors listed have made a substantial, direct, and intellectual contribution to the work, and approved it for publication.

## Conflict of Interest

The authors declare that the research was conducted in the absence of any commercial or financial relationships that could be construed as a potential conflict of interest.

## Publisher’s Note

All claims expressed in this article are solely those of the authors and do not necessarily represent those of their affiliated organizations, or those of the publisher, the editors and the reviewers. Any product that may be evaluated in this article, or claim that may be made by its manufacturer, is not guaranteed or endorsed by the publisher.
